# Grape Seed Extract Polyphenols Improve Resistance Artery Function in Pregnant eNOS^–/–^ Mice

**DOI:** 10.3389/fphys.2020.588000

**Published:** 2020-11-06

**Authors:** Teresa Tropea, Susan L. Greenwood, Colin P. Sibley, Elizabeth C. Cottrell

**Affiliations:** ^1^Division of Developmental Biology and Medicine, Faculty of Biology, Medicine and Health, Maternal and Fetal Health Research Centre, University of Manchester, Manchester, United Kingdom; ^2^Manchester Academic Health Science Centre, Manchester University NHS Foundation Trust, St. Mary’s Hospital, Manchester, United Kingdom

**Keywords:** resistance artery, pregnancy, hypertension, polyphenols, grape seed

## Abstract

Hypertension during pregnancy is a leading cause of maternal and fetal morbidity and mortality worldwide, increasing the risk of complications including preeclampsia, intracerebral hemorrhage and fetal growth restriction. Increased oxidative stress is known to contribute to poor vascular function; however, trials of antioxidant supplementation have raised concerns about fetal outcomes, including risk of low birthweight. Grape seed extract polyphenols (GSEP) have been suggested to promote cardiovascular protection, at least in part through antioxidant actions. We tested the hypothesis that administration of GSEP during pregnancy would reduce oxidative stress and improve resistance artery function with no detrimental effects on fetal growth, in an established model of maternal hypertension associated with vascular dysfunction, the endothelial NO synthase knockout (eNOS^–/–^) mouse. Pregnant C57BL/6J (WT) and eNOS^–/–^ mice received either GSEP (200 mg/kg/day) or drinking water, between gestational (GD) day 10.5 and GD18.5. At GD17.5, maternal systolic blood pressure (SBP) was measured; at GD18.5, maternal malondialdehyde (MDA) concentrations, vascular function of aortic, mesenteric, uterine and posterior cerebral arteries was assessed, and fetal outcome evaluated. GSEP reduced maternal SBP (*P* < 0.01) and plasma MDA concentrations (*P* < 0.01) in eNOS^–/–^ mice. Whilst there was no effect of GSEP on vascular reactivity of aortas, GSEP improved endothelial-dependent relaxation in mesenteric and uterine arteries of eNOS^–/–^ mice (*P* < 0.05 and *P* < 0.001, respectively) and normalized lumen diameters of pressurized posterior cerebral arteries in eNOS^–/–^ mice (*P* < 0.001). Supplementation with GSEP had no effect in WT mice and did not affect fetal outcomes in either genotype. Our data suggest that GSEP improve resistance artery function, potentially through antioxidant actions, and provide a basis to further investigate these beneficial effects including in the prevention of intracerebral hemorrhage. Maternal supplementation with GSEP may be a safe intervention to improve outcomes in pregnancies associated with hypertension and vascular dysfunction.

## Introduction

Pregnancies complicated by chronic hypertension have an increased risk of adverse perinatal outcomes (Gilbert et al., 2007; Chappell et al., 2008), including fetal growth restriction (FGR), preterm delivery, and perinatal death (Jain, 1997; Sibai et al., 1998; Bramham et al., 2014), as well as life-threatening maternal consequences, such as preeclampsia (PE), superimposed PE, eclampsia, cerebral hemorrhage, and maternal death (Sibai, 2002; Bateman et al., 2006; Bellamy et al., 2007; Moodley, 2008). By definition, chronic hypertension is the diagnosis of elevated blood pressure of ≥140 mmHg systolic and/or 90 mm Hg diastolic, before pregnancy or before 20 weeks of gestation (Sibai, 2002). Chronic hypertension affects 1–5% of pregnancies (Haddad and Sibai, 1999; Livingston and Sibai, 2001; Bramham et al., 2014; Sutton et al., 2018), with prevalence increasing with increasing maternal age (Maducolil et al., 2020).

Amongst all of the detrimental outcomes, intracerebral hemorrhage is the main cause of maternal mortality in hypertensive disorders of pregnancy worldwide (Ascanio et al., 2019; Conti-Ramsden et al., 2019). Analysis of the global burden of hypertension in pregnancy estimates the highest prevalence rates to be in developing countries (Noubiap et al., 2019).

In the absence of complications, normotensive pregnant women undergo a physiological decrease in blood pressure toward the end of the first trimester, secondary to increased vasodilation; women with chronic hypertension do not always experience this maternal cardiovascular adaptation (Seely and Ecker, 2014). During pregnancy, the endothelium plays a major role in regulating changes in vascular smooth muscle tone in the uterine and systemic circulation, by balancing the release of both endogenous vasodilators and vasoconstrictors (Tanbe and Khalil, 2010). Abnormalities in the bioavailability and bioactivity of these factors, particularly the vasodilator nitric oxide (NO), impairs endothelium-dependent vasodilation, which results in endothelial dysfunction (Albrecht et al., 2003; Landmesser et al., 2004). Moreover, the high nutrient and energy requirements for fetal growth makes the placenta a highly metabolically active organ (Vaughan and Fowden, 2016), which generates reactive oxygen species (ROS) that contribute to maternal systemic oxidative stress (Myatt, 2010). Excessive ROS may cause or exacerbate endothelial dysfunction (Lum and Roebuck, 2001) and predispose to the development of subsequent maternal cardiovascular disorders, including PE (Miranda Guisado et al., 2012; Schoots et al., 2018; Chiarello et al., 2020; San Juan-Reyes et al., 2020).

In addition to antihypertensive medications to manage maternal blood pressure, interventions aimed at targeting the increased oxidative stress may be able to reduce secondary consequences and prevent worsening of maternal disease. Evidence from some clinical trials (Poston et al., 2006) do not encourage maternal supplementation with antioxidants such as vitamin C and vitamin E, as these interventions have not been shown to improve maternal health outcomes, and have also been associated with increased rates of low-birthweight babies. Trials aimed at improving pregnancy outcomes in other high-risk groups have shown benefits with the use of supplements containing high levels of polyphenols. Maternal administration of a catechin-rich dietary supplement (epigallocatechin 3-gallate, EGCG), has been demonstrated to improve both maternal and neonatal outcomes in pregnancies affected by gestational diabetes mellitus, including neonatal weight at birth (Zhang et al., 2017), and data from a recent randomized placebo-controlled, double-blind pilot study (Matthews et al., 2019), have provided preliminary evidence of *in utero* neuroprotective effects of polyphenol-rich pomegranate juice, in newborns from FGR pregnancies. These approaches may be of benefit in hypertensive pregnancies, though data are currently lacking.

Grape seed extract polyphenols (GSEP), a complex mixture mainly composed of polymers of catechin (Shi et al., 2003), have a great potential for protection against oxidative stress (Bagchi et al., 2000). Evidence from previous studies in non-pregnant humans, non-pregnant and pregnant animals suggests that commercially available GSEP provide a natural source of antioxidants, which can protect endothelial function and reduce blood pressure (Oueslati et al., 2016; Park et al., 2016; Pons et al., 2016; Zhu et al., 2018). These beneficial effects, in addition to improved cardiovascular and kidney remodeling, have been previously demonstrated in pregnant mice, using the Nω-Nitro-l-arginine methyl ester (L-NAME)-induced hypertension model (Zhu et al., 2018). However, to date there have been no reports on the effects of GSEP supplementation in pregnancy on maternal resistance artery (diameter < 300 μm) function or pregnancy outcomes, in an animal model of chronic hypertension.

Hence, this study addressed the hypothesis that administration of GSEP during pregnancy would reduce oxidative stress and improve resistance artery function to protect the maternal vascular system, in an established model of maternal hypertension associated with vascular dysfunction, the endothelial NO synthase knockout (eNOS^–/–^) mouse (Kusinski et al., 2012).

## Materials and Methods

### Experimental Animals

The present study was performed in accordance with the UK Animals (Scientific Procedures) Act of 1986, under Home Office Project Licenses PPL 40/3385 and P9755892D. All protocols were approved by the Local Animal Welfare and Ethical Review Board of the University of Manchester. Endothelial NO synthase knockout (eNOS^–/–^) mice (stock number 002684), were purchased from Jackson Laboratories (Bar Harbor, ME, United States). C57BL/6J mice (Charles River Laboratories), the background strain for eNOS^–/–^ mice, were used as wildtype (WT) control mice. All animals were housed in individually ventilated cages maintained under a constant 12 h light/dark cycle at 21–23°C, and had free access to food (BK001 diet, Special Dietary Services, United Kingdom) and water (Hydropac, Lab products Inc., Seaford, DE, United States). Female mice (10–18 weeks old) were mated overnight with genotype-matched male mice and the presence of a copulation plug on the following morning was defined as gestational day (GD) 0.5 (estimated term, GD19.5). At GD10.5, mice were weighed and randomly assigned to receive either grape seed extract polyphenols (GSEP; Meganatural-BP Grape Seed Extract, Polyphenolics, Madera, CA)^1^, 200 mg/kg/day solubilized in the drinking water, to deliver an amount equivalent to a human dose of 1 g/d (according to Food and Drug Administration criteria for converting drug equivalent dosages across species), or drinking water alone for the remainder of pregnancy. Fresh bottles were made up daily and fluid consumption was recorded. Supplementation with GSEP was started at mid-gestation, when the mature mouse placenta is formed, but continues to grow (∼GD10.5; Woods et al., 2018). Additionally, this gestational age is analogous to the second trimester of human pregnancy, when early FGR can be diagnosed and therapeutic intervention initiated. At GD18.5, animals were sacrificed by cervical dislocation; maternal blood samples were collected and maternal and fetal tissues harvested.

### Systolic Blood Pressure and Heart Rate Measurements

Systolic blood pressure (SBP) and heart rate (HR) were determined in a subset of pregnant mice prior to assignment of the treatments and at GD17.5, using a validated non-invasive tail-cuff method (LE5001; Pan Lab, Spain). A small rodent restraining tube was placed on a thermo-pad mat. Mice were acclimatized to reduce stress before measurements were taken and once in the restraining tube, they were secured with the tail exposed for the tail cuff occlusion device and pulse transducer. SBP and HR were recorded 5–10 min after placing the devices on the tail. A total of 12–18 readings was taken within approximately 45 min and averaged for each individual mouse.

### Measurement of Lipid Peroxidation in Maternal Plasma

Lipid peroxidation was evaluated as an indicator of oxidative stress in maternal plasma. Blood samples were collected into capillary tubes (Microvette CB 300, Sarstedt) and immediately centrifuged at 5,000 rpm for 5 min; plasma was stored at −80°C until thiobarbituric acid−reactive substances (TBARS) assay was performed according to the manufacturer’s instructions (Cayman, cat. no 700870). Malondialdehyde (MDA) is a by-product of lipid peroxidation that reacts with thiobarbituric acid (TBA). Herein, the TBARS assay was used to measure MDA−TBA adduct, thus providing an estimate of MDA concentrations in maternal plasma (expressed in nM).

### Wire Myography

Maternal aorta, mesentery and uterus were rapidly removed and collected in ice-cold-physiological saline solution (PSS; in mM, 117 NaCl, 25 NaHCO_3_, 4.69 KCl, 2.4 MgSO_4_, 1.6 CaCl_2_, 1.18 KH_2_PO_4_, 6.05 glucose, 0.034 EDTA; pH 7.4) for *ex vivo* assessment of tone generation, using wire myography. Abdominal aortic, third-order mesenteric arteries and main branch uterine arteries were dissected free from perivascular connective and adipose tissue in ice-cold PSS. Vessels were cut in short segments (approximately 2 mm in length), mounted on two 40 μm steel wires in a myograph chamber (Model 620 M, Danish MyoTechnologies, Denmark) and immersed in 6 mL of 5%CO_2_/20% oxygen/75% nitrogen gassed PSS, at 37°C. Vessels were then normalized to 0.9 of luminal pressure (L)_13.3_ kPa, through a series of stepwise stretches to determine their optimal resting tension (Mulvany and Halpern, 1977) and equilibrated for 20 min. Post-equilibration, vascular segments underwent two separate exposures to a depolarizing solution (KPSS; 120 mM KCl in PSS, equimolar substitution of KCl for NaCl). After washing with PSS, a concentration-response curve to the thromboxane mimetic U46619 (10^–10^ – 2 × 10^–6^ M, Cayman Chemicals) was assessed and used to obtain the EC_80_ concentration of U46619. Endothelium-dependent relaxation to acetylcholine (ACh, 10^–10^–10^–5^ M) and endothelium-independent relaxation to the NO donor, sodium nitroprusside (SNP, 10^–10^–10^–5^ M) were assessed in vessels pre-constricted with an EC_80_ dose of U46619. Vessel tension produced by maximal depolarization to KPSS and concentration-response curve to U46619 was expressed as active effective pressure in kPa; Cmax was the maximum effect of the dose-response constriction with U46619. Concentration-response relaxation to ACh and SNP was expressed as a remaining percentage from the level of pre-constriction achieved with an EC_80_ dose of U46619. Maximum relaxation (Rmax) to ACh and SNP was expressed as a percentage of the maximum effect of the dose-response. Effects on the sensitivity (the molar concentration of U46619, ACh and SNP causing 50% of the maximal response), were expressed as logarithm (Log EC_50_).

Dose-response effects of the extract to induce vasorelaxation were confirmed in a separate set of wire myography experiments performed in human chorionic plate blood vessels, to confirm bioreactivity of the batch of GSEP, prior to the commencement of the present study (data not shown).

### Pressure Myography

Maternal brains were quickly removed and placed in ice-cold PSS. Second-order branches of posterior cerebral arteries were gently dissected and cleared of connective tissue. Arterial segments (approximately 2 mm long) were mounted on two glass cannulas within a chamber for small-vessel pressure myography studies, with both ends secured, to assess diameter changes as previously described (Tropea et al., 2015). Before starting the experiment, intraluminal pressure was increased through the proximal cannula using a servo-null pressure system (Living Systems Instrumentation) to remove the intraluminal content; then, the distal cannula was closed off to avoid any loss of pressure. Arteries with no constant pressure were discarded. Lumen diameter was visualized by an inverted microscope connected to a CCD camera and a monitor and was continuously recorded with a data-acquisition software (LabChart, AD Instruments).

All arteries were continuously superfused with warmed PSS at 37°C, aerated with 5%CO_2_/20% oxygen/75% nitrogen and pressurized to 50 mmHg. Following 45 min of equilibration under no-flow conditions, artery viability was evaluated by superfusing with KPSS. After washing with PSS, changes in lumen diameter were monitored by increasing intraluminal pressure from 10 to 110 mmHg (20 mmHg increments), allowing arteries to equilibrate to steady-state at each pressure.

Passive measurements of lumen diameter, distensibility and wall stress were obtained in fully dilated arteries by superfusing with PSS containing diltiazem (10^–5^ M) and papaverine (10^–4^ M) at each pressure, as previously described (Cipolla et al., 2006).

### Drugs and Chemicals

Chemicals and reagents used in the present study were purchased from Sigma Aldrich, unless otherwise stated.

### Data Analysis

Data are expressed as mean ± SD (for parametric data) or median and range (for non-parametric data; fluid intake, litter size, resorptions) and N is the number of mice used. Non-parametric data were transformed prior to analysis using 2-way ANOVA, which included genotype and GSEP treatment as independent variables, with Sidak’s *post-hoc* testing and adjustment for multiple comparisons, as appropriate, performed using Prism 8 software (GraphPad Software Inc., La Jolla, CA). Differences were considered significant at *P* < 0.05.

## Results

### GSEP Supplementation Had No Effect on Fluid Consumption or Maternal Body Weight in Pregnant Mice

There were no differences in maternal fluid intake following GSEP treatment compared with water controls in either WT or eNOS^–/–^ mice [WT H_2_O, 9.8 (6.1–25.7); WT GSEP, 8.1 (5.8–21.8); eNOS^–/–^ H_2_O, 8.6 (6.1–11.4); eNOS^–/–^ GSEP, 9.9 (7.5–11.5); mL/day]. Maternal body weight (BW) was not different between genotypes at GD0.5, or at GD10.5, before the commencement of the treatment (Table 1). WT mice were significantly heavier than eNOS^–/–^ mice at GD18.5 (*P* < 0.01, Table 1), but the treatment with GSEP had no effect on maternal BW (Table 1).

**TABLE 1:** Maternal body weight.

Genotype	WT		eNOS^–/–^		Effects		
Treatment	H_2_O	GSEP	H_2_O	GSEP	Treat	Gen	Int
BW at GD0.5 (g)	21.5 ± 1.6	21.3 ± 1.0	19.9 ± 1.4	19.9 ± 1.0	NS	NS	NS
BW at GD10.5 (g)	25.6 ± 2.4	25.4 ± 1.0	23.6 ± 1.6	23.6 ± 1.2	NS	NS	NS
BW at GD18.5 (g)	38.5 ± 3.6	38.7 ± 2.6	34.9 ± 4.1	34.1 ± 2.2	NS	##	NS

*Data are expressed as mean ± SD. Treat, effect of treatment; Gen, effect of genotype; Int, interaction between genotype and treatment. ^##^P < 0.01 WT vs. eNOS^–/–^; NS, not significant. N = 8–11 dams per group.*

### Treatment With GSEP Reduced Maternal Systolic Blood Pressure and Had No Effect on Heart Rate in eNOS^–/–^ Mice

**FIGURE 1:**
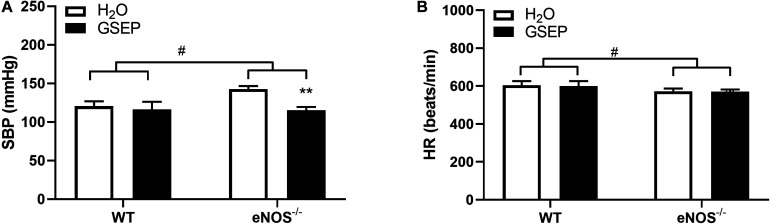
GSEP supplementation lowers systolic blood pressure without affecting heart rate in eNOS^–/–^ mice at GD17.5. In eNOS^–/–^ mice, systolic blood pressure (SBP) was significantly higher compared with WT animals, and was reduced by supplementation with GSEP **(A)**. Heart rate (HR) was significantly lower in eNOS^–/–^ mice compared with WT animals but was unaffected by GSEP treatment **(B)**. ^#^*P* < 0.05 eNOS^–/–^ vs. WT; **P < 0.01 GSEP vs. H_2_O. *N* = 3–4 dams per group.

Prior to the start of treatment at GD10.5, eNOS^–/–^ mice showed significantly higher SBP (WT, 115.0 ± 4.7, eNOS^–/–^, 141.6 ± 10.1 mmHg; *P* < 0.001) and lower HR (WT, 635.9 ± 40.0, eNOS^–/–^, 554.9 ± 64.5 beats/min; *P* < 0.01) compared with WT. Supplementation with GSEP significantly lowered maternal SBP in eNOS^–/–^ mice (effect of treatment, *P* < 0.01; Figure 1A), but did not change SBP in WT animals, and had no effect on HR in either genotype (Figure 1B) at GD17.5. A genotype effect on HR remained at GD17.5 (*P* < 0.05; Figure 1B).

### GSEP Supplementation Reduced Levels of Oxidative Stress in Maternal Plasma of eNOS^–/–^ Mice

**FIGURE 2:**
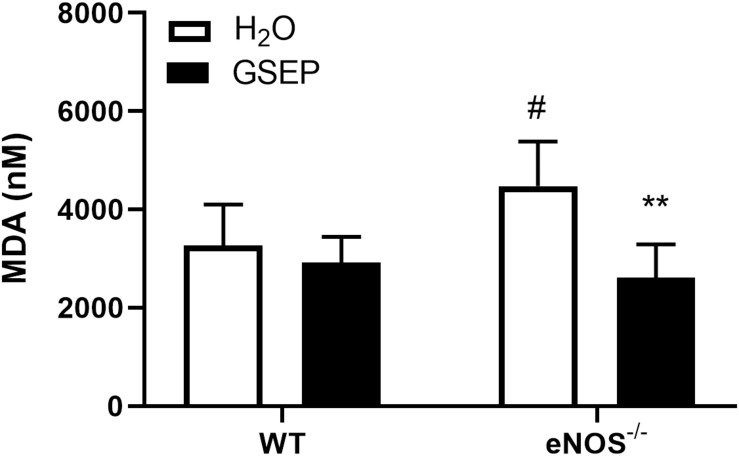
Treatment with GSEP decreases oxidative stress in maternal plasma of eNOS^–/–^ mice. Concentrations of malondialdehyde (MDA) were significantly higher in maternal plasma of eNOS^–/–^ mice compared with WT H_2_O mice and were significantly reduced in eNOS^–/–^ mice treated with GSEP. ^#^*P* < 0.05 eNOS^–/–^ vs. WT H_2_O; **P < 0.01 GSEP vs. H_2_O. *N* = 5–6 dams per group.

MDA concentrations detected in maternal plasma were significantly increased in eNOS^–/–^ mice compared with WT H_2_O mice (*P* < 0.05; Figure 2). Supplementation with GSEP had significant treatment (*P* < 0.01; Figure 2) and interaction effects (*P* < 0.05; Figure 2) on MDA levels, significantly reducing the marker of lipid peroxidation in eNOS^–/–^ mice only (*P* < 0.01; Figure 2).

### GSEP Supplementation Induced Changes in Vascular Reactivity of Maternal Aortic, Mesenteric, and Uterine Arteries of eNOS^–/–^ Mice

**FIGURE 3:**
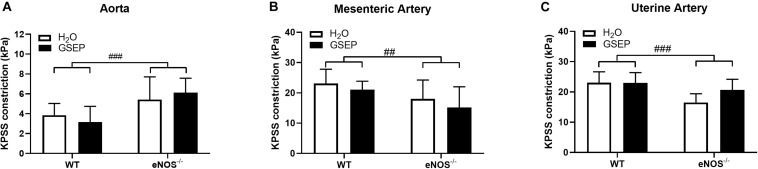
Supplementation with GSEP has no effect on maximal smooth muscle depolarization. Treatment with GSEP did not alter constriction of either aortas **(A)**, mesenteric **(B)**, or uterine **(C)** arteries in response to a maximal smooth muscle depolarization induced by KPSS in WT or eNOS^–/–^ mice. ^##^*P* < 0.01, ^###^*P* < 0.001 eNOS^–/–^ vs. WT. *N* = 6–11 dams per group.

Following exposure to the maximally depolarizing potassium solution, aortas of eNOS^–/–^ mice developed greater tension than WT (effect of genotype, *P* < 0.001; Figure 3A), whereas mesenteric and uterine arteries showed reduced vascular contractility to KPSS compared with WT (effect of genotype, *P* < 0.01, *P* < 0.001, respectively; Figures 3B,C); treatment with GSEP had no effect on KPSS constriction (Figure 3).

**FIGURE 4:**
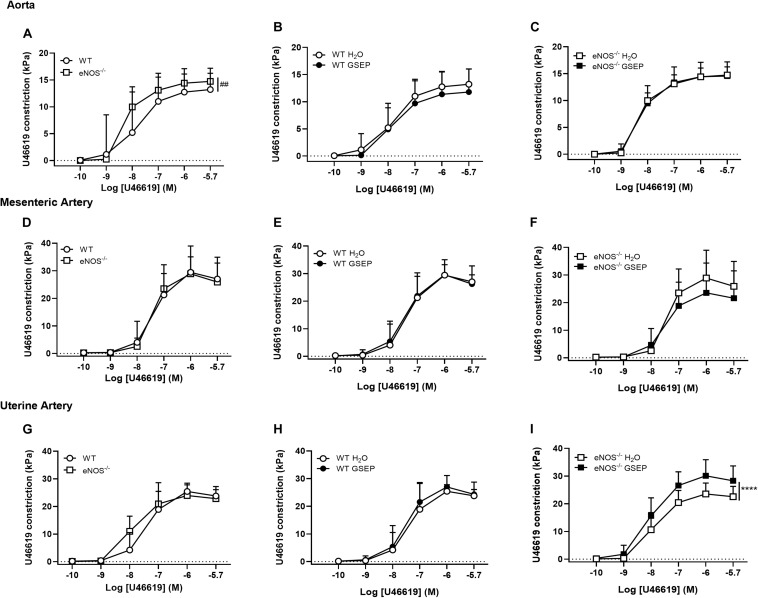
GSEP supplementation increases thromboxane mimetic-induced constriction of uterine arteries in eNOS^–/–^ mice. Aortas of eNOS^–/–^ mice **(A)**, but not mesenteric **(D)** or uterine arteries **(G)**, showed significantly higher constriction to U46619 compared with WT controls. Maternal GSEP supplementation did not affect constriction in response to U46619 of either aortas **(B,C)** or mesenteric arteries **(E,F)** in WT or eNOS^–/–^ mice. In uterine arteries, GSEP treatment had no effect on U46619-induced constriction in WT **(H)** but significantly increased vasoconstriction in eNOS^–/–^ mice **(I)**. ^##^*P* < 0.01 eNOS^–/–^ vs. WT; ****P < 0.0001 GSEP vs. H_2_O. *N* = 6–11 dams per group.

Both constriction and sensitivity to the thromboxane mimetic U46619 were higher in aortas of eNOS^–/–^ mice compared with WT mice (Cmax: WT, 13.3 ± 2.8, eNOS^–/–^, 14.7 ± 2.5 kPa; effect of genotype, *P* = 0.053; Log EC_50_: WT, −7.91 ± 0.6, eNOS^–/–^, −8.260 ± 0.1; effect of genotype, *P* < 0.05; Figure 4A); there were no differences in mesenteric arteries (Figure 4D), whereas uterine arteries of eNOS^–/–^ mice were more sensitive than WT mice to U46619 (Log EC_50_: WT, −7.40 ± 0.6, eNOS^–/–^, −7.92 ± 0.4; effect of genotype, *P* < 0.01; Figure 4G). Treatment with GSEP did not have a significant effect on constriction to U46619 in either aortas (Figures 4B,C) or mesenteric arteries (Figures 4E,F) of both genotypes, or in uterine arteries of WT animals (Figure 4H), but significantly increased vascular reactivity to the thromboxane mimetic in uterine arteries of eNOS^–/–^ mice compared with H_2_O controls (*P* < 0.0001; Figure 4I).

**FIGURE 5:**
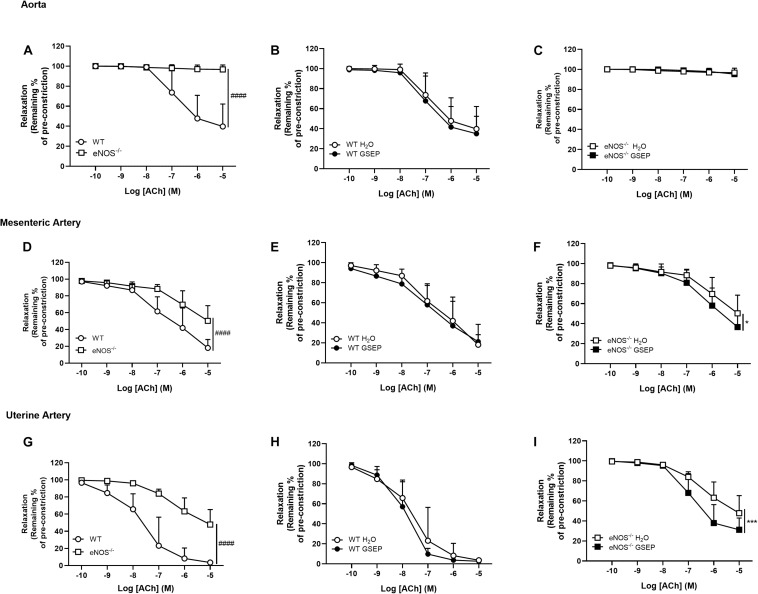
GSEP supplementation improves endothelium-dependent relaxation of mesenteric and uterine arteries in eNOS^–/–^ mice. In eNOS^–/–^ mice, aortas did not relax in response to ACh **(A,C)** and endothelium-dependent relaxation was significantly reduced in both mesenteric **(D)** and uterine **(G)** arteries compared with WT controls. Supplementation with GSEP had no effect on endothelium-dependent relaxation to ACh in aortas of WT mice **(B)**. In both mesenteric and uterine arteries, relaxation to ACh was unaffected by GSEP in WT mice (**E,H**, respectively) but was significantly enhanced in eNOS^–/–^ mice (**F,I**, respectively). ^####^*P* < 0.0001 eNOS^–/–^ vs. WT; *P < 0.05, ***P < 0.001 GSEP vs. H_2_O. *N* = 6–11 dams per group.

There was no endothelium-dependent relaxation to ACh in aortas of eNOS^–/–^ mice compared with WT mice (Figure 5A) and treatment with GSEP did not affect relaxation to ACh in aortas of both WT and eNOS^–/–^ mice (Figures 5B,C). Endothelium-dependent relaxation was significantly reduced in both mesenteric and uterine arteries of eNOS^–/–^ mice compared with WT mice (Rmax: mesenteric arteries, WT, 81.9 ± 10.0, eNOS^–/–^, 49.7 ± 18.1%; uterine arteries, WT, 96.4 ± 2.2, eNOS^–/–^, 52.2 ± 17.5%; effect of genotype, *P* < 0.0001 both; Figures 5D,G), and sensitivity was lower only in uterine arteries of eNOS^–/–^ mice compared with WT mice (Log EC_50_: WT, −7.54 ± 0.7, eNOS^–/–^, −6.41 ± 0.4; effect of genotype, *P* < 0.0001; Figure 5G). Treatment with GSEP had no effect on ACh responses in either mesenteric or uterine arteries of WT animals (Figures 5E,H), but significantly enhanced endothelium-dependent relaxation in both of these vascular beds in eNOS^–/–^ mice (*P* < 0.05, *P* < 0.001; mesenteric and uterine arteries, respectively; Figures 5F,I).

**FIGURE 6:**
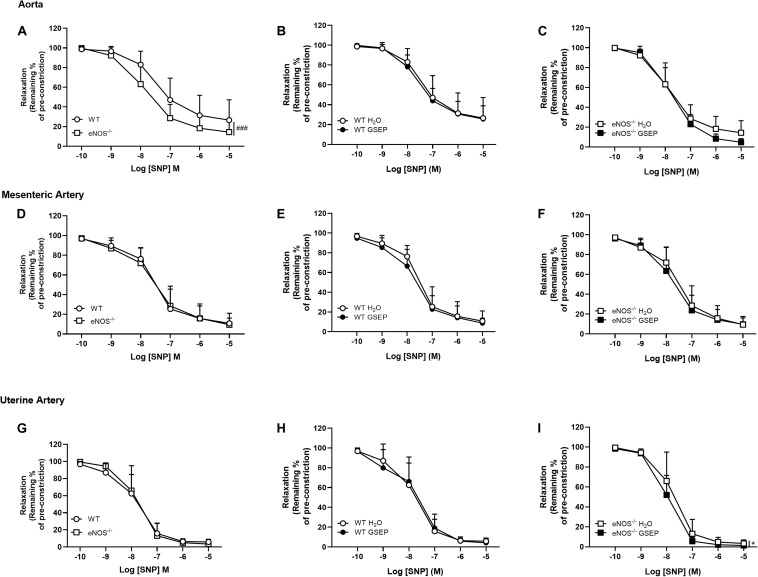
Treatment with GSEP enhances endothelium-independent relaxation of uterine arteries in eNOS^–/–^ mice. Endothelium-independent relaxation in response to SNP was significantly increased in aortas of eNOS^–/–^ mice compared with WT controls **(A)**, but did not change between genotypes in either mesenteric **(D)** or uterine arteries **(G)**. Supplementation with GSEP had no effect on responses to SNP in either aortas **(B,C)** or mesenteric arteries **(E,F)** from WT and eNOS^–/–^ mice. In uterine arteries, the treatment had no effect in WT **(H)** but significantly improved relaxation to SNP in eNOS^–/–^ mice **(I)**. ^###^*P* < 0.001 eNOS^–/–^ vs. WT; *P < 0.05 GSEP vs. H_2_O. *N* = 6–11 dams per group.

Endothelium-independent relaxation and sensitivity to the NO donor SNP were significantly increased in aortas of eNOS^–/–^ mice compared with WT mice (Rmax: WT, 73.4 ± 20.7, eNOS^–/–^, 85.6 ± 12.1%; effect of genotype, *P* < 0.01; Log EC_50_: WT, −7.43 ± 0.4, eNOS^–/–^, −7.86 ± 0.4; effect of genotype, *P* < 0.05; Figure 6A). There were no genotype-differences in response to SNP in either mesenteric or uterine arteries (Figures 6D,G). Treatment with GSEP did not affect aortas of either genotype (Figures 6B,C), or uterine arteries of WT controls (Figure 6H). GSEP had a slight but significant effect to enhance vascular smooth muscle sensitivity to SNP in mesenteric arteries of WT and eNOS^–/–^ mice (Log EC_50_: WT H_2_O, −7.52 ± 0.3, WT GSEP, −7.75 ± 0.3, eNOS^–/–^ H_2_O, −7.51 ± 0.4, eNOS^–/–^ GSEP, −7.77 ± 0.4; effect of treatment, *P* < 0.05; Figures 6E,F), and relaxation in response to SNP in uterine arteries of eNOS^–/–^ mice (*P* < 0.05; Figure 6I).

### GSEP Supplementation Prevented Pressure-Induced Increase in Diameter of Posterior Cerebral Arteries in eNOS^–/–^ Mice

**FIGURE 7:**
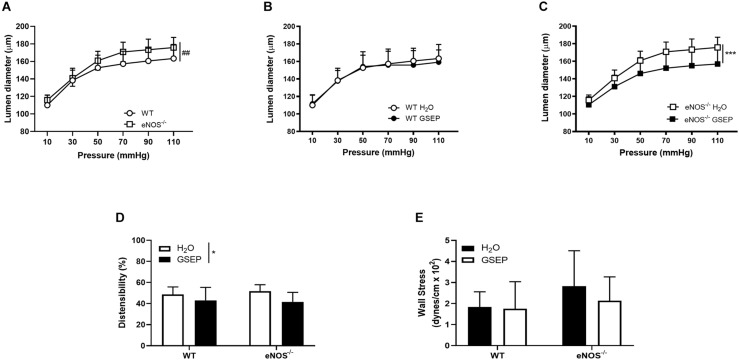
Treatment with GSEP normalizes diameter of posterior cerebral arteries in eNOS^–/–^ mice. Increasing intraluminal pressure (10–110 mmHg) significantly expanded lumen diameters of fully dilated posterior cerebral arteries of eNOS^–/–^ mice compared with WT controls **(A)**. Treatment with GSEP had no effect on lumen diameters of WT mice **(B)**, but significantly reduced diameters in eNOS^–/–^ mice **(C)**. Distensibility **(D)** but not circumferential wall stress **(E)** at 110 mmHg was significantly affected by the treatment. ^##^*P* < 0.01 eNOS^–/–^ vs. WT;*P < 0.05; ***P < 0.001 GSEP vs. H_2_O. *N* = 6–9 dams per group.

A stepwise increase in intraluminal pressure (10–110 mmHg), revealed that posterior cerebral arteries of eNOS^–/–^ mice display larger lumen diameters than WT mice (effect of genotype on steady-state diameters across pressure range, *P* < 0.01; Figure 7A). Maternal treatment with GSEP did not affect lumen diameters of WT mice (Figure 7B), but significantly reduced diameters of posterior cerebral arteries of eNOS^–/–^ mice (*P* < 0.001; Figure 7C). GSEP reduced distensibility (overall effect of treatment, *P* < 0.05; Figure 7D), whereas wall stress was unaltered (Figure 7E), at the highest pressure assessed (110 mmHg).

### Supplementation With GSEP Had No Effect on Pregnancy Outcomes

eNOS^–/–^ mice had significantly smaller fetuses compared with WT mice (*P* < 0.0001; Table 2). There were no significant differences in placental weights, litter size or number of resorptions between genotypes, and maternal treatment with GSEP did not alter fetal outcomes in WT or eNOS^–/–^ mice at GD18.5 (Table 2).

**TABLE 2:** Pregnancy outcomes.

Genotype	WT		eNOS^–/–^		Effects		
Treatment	H_2_O	GSEP	H_2_O	GSEP	Treat	Gen	Int
Fetal weight (g)	1.14 ± 0.05	1.14 ± 0.05	0.95 ± 0.09	0.99 ± 0.07	NS	####	NS
Placental weight (mg)	80.6 ± 3.9	80.4 ± 4.2	80.9 ± 6.9	81.3 ± 4.9	NS	NS	NS
Litter size (fetuses/dam)	8.0 (5.0–11.0)	8.0 (1.0–10.0)	8.0 (5.0–9.0)	7.0 (4.0–9.0)	NS	NS	NS
Resorptions (number/litter)	2.0 (0.0–3.0)	1.0 (0.0–4.0)	0.5 (0.0–2.0)	0.5 (0.0–3.0)	NS	*P* = 0.0651	NS

*Fetal and placenta weights are expressed as mean ± SD. Litter size and number of resorptions are expressed as median and range. Treat, effect of treatment; Gen, effect of genotype; Int, interaction between genotype and treatment. ^####^P < 0.0001 WT vs. eNOS^–/–^; NS, not significant. N = 6–11 dams per group.*

## Discussion

Chronic hypertension in pregnancy is associated with significant short- and long-term adverse maternal and fetal consequences. In low−income countries, where maternal hypertension remains highly prevalent, maternal and perinatal mortality are still a major concern (Noubiap et al., 2019), and adequate primary health care and prompt management of hypertension could significantly contribute to preventing the development of the most severe outcomes (Bagga et al., 2007).

To our knowledge, this is the first study investigating whether supplementation with GSEP, a widely available and highly bioactive compound, can improve vascular function in key target blood vessels in an established animal model of chronic hypertension in pregnancy.

The main finding of this study was that treatment with GSEP from mid-pregnancy may offer a broad protection of the maternal vasculature against hypertension and oxidative stress, by improving the function of resistance arteries and lowering SBP in pregnant eNOS^–/–^ mice. The data substantially support our starting hypothesis and, importantly, these beneficial effects on maternal vascular function were not associated with any adverse fetal outcomes, suggesting this approach is safe.

Two clinical trials have already shown the potential for supplementation with GSEP to reduce blood pressure in non-pregnant patients with pre-hypertension (Park et al., 2016; Odai et al., 2019). In addition, a previous preclinical study investigated the effect of intragastric administration of GSEP in pregnancy, using the L-NAME-induced hypertensive mouse model. The authors measured SBP under anesthesia and demonstrated that treatment with GSEP reversed the hypertensive effect induced by L-NAME after 3 weeks of intervention (Zhu et al., 2018). In agreement with this finding, the current study showed a significant blood pressure-lowering effect of maternal supplementation with GSEP in pregnant eNOS^–/–^ mice.

Unlike the L-NAME-induced hypertension model, eNOS^–/–^ mice display chronic hypertension (Shesely et al., 1996) and lower HR (Kulandavelu et al., 2006) compared with WT mice. The measurements of SBP at GD17.5 in eNOS^–/–^ mice in the current study are consistent with our own data (Tropea et al., 2020) and those reported by others (Hefler et al., 2001; Poudel et al., 2013). Administration of a different polyphenol, resveratrol (3,5,4′-trihydroxystilbene), was reported to have no effect on SBP in eNOS^–/–^ mice (Poudel et al., 2013). In contrast, our recent data demonstrated that supplementation with beetroot juice significantly lowered SBP in eNOS^–/–^ mice, effects which we believe are potentially attributable to bioactive phytochemicals contained within the juice, including polyphenols and flavonoids (Tropea et al., 2020), in common with the active constituents of GSEP. The phenolic content of the extract used in the current study has been reported to be 94% and mostly made of polymers of catechin (Edirisinghe et al., 2008; Sivaprakasapillai et al., 2009), shown to provide a higher antioxidant ability compared to other commonly used antioxidants (Bagchi et al., 1997, 1998). *In vitro* studies, have shown that GSEP are a better scavenger of free radicals, exhibiting 2–4 times more potent scavenging activity, than vitamin C and vitamin E (Bagchi et al., 1997). Similarly, *in vivo* studies have demonstrated that GSEP provide a greater protection against lipid peroxidation and DNA fragmentation compared with vitamin C, vitamin E and β-carotene, in hepatic and brain tissues of the 12-O-tetradecanoylphorbol-13-acetate (TPA)-induced oxidative damage mouse model (Bagchi et al., 1998).

Normal pregnancy is associated with oxidative stress (Chiarello et al., 2020), as increased ROS production plays a key role in signaling pathways that promote trophoblast invasion and placenta angiogenesis (Pereira et al., 2015). However, in addition to placental oxidative stress and impaired placental function (Myatt and Cui, 2004; Burton, 2009), an excessive production of ROS has been associated with the pathophysiology of many maternal hypertensive pregnancy-related complications, such as PE (Myatt and Cui, 2004; Burton and Jauniaux, 2011; Matsubara et al., 2015; Chiarello et al., 2020; San Juan-Reyes et al., 2020). In agreement with recently published data in the L-NAME mouse model (Zhu et al., 2018), we found that circulating concentrations of MDA were increased in the maternal plasma of eNOS^–/–^ mice compared with WT control groups, and that treatment with GSEP significantly reduced this marker of lipid peroxidation in the hypertensive strain. One significant limitation of this study is mouse plasma volume availability, as investigation of several relevant biomarkers in addition to MDA, are needed to address a complete evaluation of oxidative stress in both maternal and fetal samples.

MDA is a major metabolite of lipid peroxide breakdown and one of the first biomarkers of oxidative stress found in the maternal circulation of women with PE (Myatt and Cui, 2004; Kaur et al., 2008; Poston et al., 2011; Haram et al., 2019). Thus, the similarities of findings in hypertensive animal models, such as those used in the present study, with those described in pregnant women (Myatt and Cui, 2004; Kaur et al., 2008; Poston et al., 2011) support the use of these animal models in translational pregnancy research.

There is still very little convincing evidence that supplementation with known antioxidants can elicit potential benefit in pregnant women with a range of clinical risk factors. Despite promising effects in a pilot trial (Chappell et al., 2002), subsequent studies evaluating the effect of supplementation with vitamins C and E in women at high-risk of developing PE not only failed to prevent the disease (Poston et al., 2006), but importantly, revealed increased rate of low-birthweight babies in the treatment arm of the study, thus precluding the use of this particular antioxidant strategy in pregnancy (Poston et al., 2006). Of note, pro-oxidant activity of vitamins C and E has been demonstrated at high doses (Pearson et al., 2006; Varadharaj et al., 2006), emphasizing the importance of dose selection when considering any antioxidant strategy. However, a wide variety of dietary sources of natural antioxidants, including polyphenols, is underexplored and investigation of their effects in pregnancy is currently a key research area. A recent randomized controlled pilot study has shown potential neuroprotective effects of maternal pomegranate juice supplementation, which is also rich in antioxidants and bioactive polyphenols, in FGR newborns at risk for hypoxic-ischemic injury (Matthews et al., 2019). A double−blind randomized controlled trial using EGCG as an intervention in pregnant women with gestational diabetes mellitus, reported therapeutic effects of this natural catechin-rich compound for both maternal and neonatal complications, such as improved low-birthweight and lower number of infants with hypoglycemia (Zhang et al., 2017). Similarly, cardioprotective effects against doxorubicin-mediated myocyte dysfunction were seen in cardiomyocytes of neonatal rats following catechin-rich polyphenolic supplementation (Li et al., 2010).

A growing body of evidence from clinical trials has shown beneficial effects of supplementation with GSEP to reduce oxidized LDL, aiming to lower the risk of cardiovascular disorders in non-pregnant patients (Preuss et al., 2000; Sano et al., 2007; Razavi et al., 2013), and supplementation with GSEP has been demonstrated to reduce brain lipid peroxidation and provide neuroprotection in newborns from a rat hypoxia-ischemic brain injury model (Feng et al., 2005). To the best of our knowledge, there is no report of low birthweight or developmental toxic effects following maternal supplementation with GSEP (Zhu et al., 2018; Althali et al., 2019) or high levels of catechin in animal models (Lesser et al., 2015).

Fetuses of eNOS^–/–^ dams display a ∼10% lower weight than those of WT and exhibit an FGR phenotype (Kusinski et al., 2012). Several treatment options have been investigated to test the potential therapeutic effect of polyphenols and other antioxidants during pregnancy in this mouse model, showing conflicting results on fetal outcomes. In a recently published work (Finn-Sell et al., 2018), maternal supplementation with pomegranate juice had detrimental effects on fetal outcomes of both WT and eNOS^–/–^ mice; whereas, treatment with resveratrol showed a trend toward an increase in fetal weight in eNOS^–/–^ mice (Poudel et al., 2013) and the antioxidant drug Tempol (4-Hydroxy-2,2,6,6-tetramethylpiperidin-1-oxyl) improved fetal growth in eNOS^–/–^ mice (Stanley et al., 2012). More recently, maternal supplementation with either melatonin (Renshall et al., 2018), or with beetroot juice (Tropea et al., 2020), had no effects on eNOS^–/–^ fetuses. In agreement with the latter data, our results showed that GSEP did not improve fetal growth in fetuses from eNOS^–/–^ dams. It appears that therapeutic interventions mostly fail to improve fetal outcomes in eNOS^–/–^ mice. The growth potential of this homozygous knockout mouse as a model of FGR may be genetically limited (Tropea et al., 2020), thus representing a key limitation for studies in which intervention aim at ameliorating the FGR phenotype.

However, there was no detrimental effect on the growth of fetuses in WT mice or any further reduction in fetal growth in eNOS^–/–^ mice. Further studies investigating the effect of these substances on postnatal growth and development in the offspring are warranted before translating potentially safe interventions into strategies for pregnant women.

### Effects of GSEP Predominate in Resistance Arteries of the Pregnant eNOS^–/–^ Mouse

Mammalian pregnancy requires physiological vascular adaptations to accommodate for the progressive increase in blood volume, while preventing the development of maternal hypertension (Osol et al., 2019). The endogenous vasodilator NO increases blood flow and regulates vascular smooth muscle tone (Moncada and Higgs, 2006) in the uterine and systemic circulation by balancing the release of both endogenous vasodilators and vasoconstrictors (Tanbe and Khalil, 2010). To investigate the potential for GSEP to alter vascular function, we explored effects of this treatment on the vascular tone of pharmacologically stimulated conduit (aorta), systemic (mesenteric) and reproductive (uterine) arteries. In agreement with previous findings in non-pregnant eNOS^–/–^ mice (Chataigneau et al., 1999; Waldron et al., 1999; Brandes et al., 2000; Lamping and Faraci, 2003), vasoconstriction and endothelium-independent relaxation were both significantly enhanced in aortas of eNOS^–/–^ compared with WT mice, whereas there was a complete absence of endothelium-dependent relaxation in these arteries. Contrary to the previous study by Zhu et al. (2018), where maternal administration of GSEP enhanced endothelium-dependent relaxation in aortas of a non-genetic model of hypertension, we did not demonstrate a similar effect of GSEP in this vascular bed.

The present study is the first to assess the effects of GSEP supplementation on resistance-sized blood vessels and demonstrates stark vascular bed differences between conduit and resistance arteries. Contrary to effects on the aorta, supplementation with GSEP significantly improved endothelium-dependent relaxation in both mesenteric and uterine arteries of eNOS^–/–^ mice.

The endothelium-dependent relaxing activity of GSEP exhibited *in vitro* in rat aortic rings has been reported to increase with the degree of proanthocyanidin polymerization (Fitzpatrick et al., 2000, 2002), and interestingly, a structure–activity relationship has been hypothesized to stimulate a pseudo laminar shear stress response in endothelial cells, through which GSEP is thought to modify vascular function (Corder et al., 2004). This new concept could suggest a pathway through which GSEP may affect the function of blood vessels.

As well as highlighting differences between conduit and resistance arteries in terms of effects of GSEP to enhance endothelium-dependent vasorelaxation, our data also illustrated that supplementation with GSEP significantly increased U46619-induced constriction in uterine arteries. As already reported for other vasoconstricting agents (D’Angelo and Osol, 1993), the higher sensitivity of uterine compared with mesenteric arteries for U46619 in eNOS^–/–^ mice (data not shown), reflects regional differences of the maternal vasculature to adapt to pregnancy (D’Angelo and Osol, 1993; Osol et al., 2019). Unlike in the uterine arteries, we observed the opposite trend in mesenteric arteries of eNOS^–/–^ mice treated with GSEP, which may underlie the SBP-lowering effect mediated by the extract in the systemic vasculature of the hypertensive strain.

The exact mechanisms through which GSEP affects these vascular beds are currently unknown, which reflects a limitation in studies investigating vascular effects of bioactive compounds through underexplored nutritional interventions. Although beyond the scope of this study, future work will focus on determining molecular pathways activated by GSEP during pregnancy.

### Effects of GSEP May Protect the Maternal Cerebral Microvasculature

Despite substantial hemodynamic changes in almost every other vascular bed, the adaptation of the cerebrovasculature to pregnancy maintains normal vascular resistance and blood flow, such that there are negligible effects on cerebral blood flow during pregnancy (Johnson and Cipolla, 2015). Impaired endothelial function and hypertension underpin the increased risk of maternal intracerebral hemorrhage in pregnant women (Shawwa et al., 2018). Under normal conditions, cerebral endothelial cells composing the blood-brain barrier provide protection from harm (Obermeier et al., 2013); however, chronic elevation of intraluminal arterial pressure can weaken the vessel wall and cause microvascular damage (Fisher, 1971). Hypertension is the most common cause of intracerebral hemorrhage (Keep et al., 2014). Indeed, as a result of the substantial increase in pressure-induced forced vasodilatation, cerebral arterioles can burst, causing intracerebral hemorrhage and blood-brain barrier disruption (Johansson et al., 1970; Keep et al., 2014).

Although rare in developed countries, intracerebral hemorrhage remains the main final cause of maternal death in hypertensive disorders of pregnancy (Ascanio et al., 2019; Conti-Ramsden et al., 2019), with the greatest incidence in the third trimester and in the puerperium (Bateman et al., 2006; Davie and O’Brien, 2008). Simulating an increase in intraluminal pressure in posterior cerebral arteries, we have shown that lumen diameter is significantly increased in arterioles of eNOS^–/–^ mice compared with those of the WT, suggesting impaired cerebral vasoregulation in this hypertensive model. Treatment with GSEP showed no effect on posterior cerebral arteries of WT mice, but in vessels from eNOS^–/–^ mice, significantly reduced lumen diameters under conditions of high intraluminal pressure. This effect of GSEP on posterior cerebral arteries could decrease transmission of pressure in the downstream microcirculation and potentially provide a protective mechanism from forced vasodilatation, by which to preserve blood brain barrier integrity. The lack of effect in WT animals suggests the safety of this intervention in pregnancy also in the cerebral vasculature. The ability of GSEP to normalize lumen diameters of posterior cerebral arteries, in addition to the positive effects on the systemic resistance arteries outlined above, is suggestive of the potential broad spectrum of protective effects of this intervention, at least in the eNOS^–/–^ mouse model. It is possible that the effects seen in this vascular bed are a direct consequence of the GSEP blood pressure-lowering properties and associated improved systemic vascular function. However, others have demonstrated alterations in extracellular matrix biological structure and function of the arterial wall in response to GSEP treatment (Ivanov et al., 2007; De Bruyne et al., 2019), which may also play a role in the eNOS^–/–^ mouse.

Taken together, our data indicate that maternal supplementation with GSEP could offer a cheap and widely available treatment to improve maternal vascular health in hypertensive pregnancies. Protective effects of GSEP against vascular dysfunction have been attributed to their antioxidant properties (Wang et al., 2010; Okudan et al., 2011; Pinna et al., 2017; Zhu et al., 2018; Sato et al., 2020). Furthermore, the treatment did not have adverse effects on fetal outcome and the potential for GSEP as a therapy for complications of chronic hypertension in pregnancy is worthy of further research.

## Conclusion

In conclusion, this study reports that maternal administration of GSEP during pregnancy leads to a reduction in SBP in eNOS^–/–^ mice, which may be mediated by the antioxidant potential of the extract to improve systemic vascular function. In addition, the effect of GSEP supplementation on cerebral arterioles indicates that GSEP may protect vascular integrity of the downstream cerebral microcirculation in the brain. The current findings warrant further investigation and require the development of preclinical models exhibiting intracerebral hemorrhage in pregnancy in which to test this intervention. Collectively, our data substantiate supplementation with GSEP as a promising treatment in pregnancies complicated by maternal hypertension. Delineating the pathways and molecular mechanisms involved in these beneficial effects will require further investigation.

## Data Availability Statement

The raw data supporting the conclusions of this article will be made available by the authors, without undue reservation, to any qualified researcher.

## Ethics Statement

The animal study was reviewed and approved by the Local Animal Welfare and Ethical Review Board of the University of Manchester.

## Author Contributions

TT conceived and designed the study, performed the research, and wrote the manuscript. SLG, CPS, and ECC contributed to the conception and design of the study and critically revised the manuscript for important intellectual content. All authors contributed to the article and approved the submitted version.

## Conflict of Interest

The authors declare that the research was conducted in the absence of any commercial or financial relationships that could be construed as a potential conflict of interest.

## Footnotes

1 http://www.polyphenolics.com/wp-content/uploads/2017/08/3-MegaNatural-BP-Brochure.pdf
